# Prospects of Polymeric Nanofibers Loaded with Essential Oils for Biomedical and Food-Packaging Applications

**DOI:** 10.3390/ijms22084017

**Published:** 2021-04-13

**Authors:** Anjum Hamid Rather, Taha Umair Wani, Rumysa Saleem Khan, Bishweshwar Pant, Mira Park, Faheem A. Sheikh

**Affiliations:** 1 Department of Nanotechnology, University of Kashmir Hazratbal, Srinagar 190006, Jammu and Kashmir, India; anjumhamid.scholar@kashmiruniversity.net (A.H.R.); wanitaha.scholar@kashmiruniversity.net (T.U.W.); rumysakhan.scholar@kashmiruniversity.net (R.S.K.); 2 Carbon Composite Energy Nanomaterials Research Center, Woosuk University, Wanju-Gun 55338, Jeollabuk-do, Korea; bisup@jbnu.ac.kr

**Keywords:** electrospinning, essential oils, food industry, wound healing, tissue engineering

## Abstract

Essential oils prevent superbug formation, which is mainly caused by the continuous use of synthetic drugs. This is a significant threat to health, the environment, and food safety. Plant extracts in the form of essential oils are good enough to destroy pests and fight bacterial infections in animals and humans. In this review article, different essential oils containing polymeric nanofibers fabricated by electrospinning are reviewed. These nanofibers containing essential oils have shown applications in biomedical applications and as food-packaging materials. This approach of delivering essential oils in nanoformulations has attracted considerable attention in the scientific community due to its low price, a considerable ratio of surface area to volume, versatility, and high yield. It is observed that the resulting nanofibers possess antimicrobial, anti-inflammatory, and antioxidant properties. Therefore, they can reduce the use of toxic synthetic drugs that are utilized in the cosmetics, medicine, and food industries. These nanofibers increase barrier properties against light, oxygen, and heat, thereby protecting and preserving the food from oxidative damage. Moreover, the nanofibers discussed are introduced with naturally derived chemical compounds in a controlled manner, which simultaneously prevents their degradation. The nanofibers loaded with different essential oils demonstrate an ability to increase the shelf-life of various food products while using them as active packaging materials.

## 1. Introduction

Phytochemicals are compounds that help plants to defend against microbes, insects, and other animals. When these phytochemicals are subjected to isolation from medicinal plants and then used in the form of plant extracts, they can have a lot of therapeutic importance [1,2,3]. The primary active compounds of the plant extracts include antioxidants and antimicrobials. However, the antimicrobial attributes of plant extracts are of significant interest nowadays [4,5,6]. The uncontrolled and massive utilization of synthetic compounds, such as antibiotics in cosmetics, medicine, pharmaceutics, and the food industry, is causing environmental concerns [7,8,9]. Interestingly, plant extracts have been largely used to treat microbial infections due to their inherent ability to treat synthetic antibiotic-resistant infections [10,11,12,13,14]. The efficient extraction and delivery of phyto-compounds (e.g., essential oils) to patients from medicinal plants is a great challenge for researchers with regard to use them as antimicrobial agents. The antimicrobial agents, e.g., essential oils, have an exclusive position to treat various diseases. However, the polymer matrix also performs a critical function in delivering the same. To achieve this goal, various polymers are used to provide a suitable vehicle system to deliver essential oils. These polymers are used primarily in the form of nanomaterials with different dimensions (e.g., 0-D, 1-D, 2-D, and 3-D). However, the nanomaterials in the form of 1-D structures; for instance, the polymeric nanofibers, are vigorously used due to their large surface-to-volume ratio compared to other nanostructures. In this regard, various techniques were previously put forward for manufacturing these nanofibers, such as freeze-drying [15,16], self-assembly [17,18,19], phase separation [19,20], template synthesis [19,21], and spinneret-based engineered parameters [16,22]. However, electrospinning is the most widely utilized technique for applications in tissue engineering [23], wound dressing [24], and drug release [25]. This method has drawn tremendous interest in the scientific community due to its low price, flexibility to tune the fiber morphology, and high yield, which make this technique industrially applicable. It produces fibers within the diameter range of few microns to the nanometer scale using high electric fields [26,27]. Moreover, essential oils, because of their antioxidant [28], antiparasitic [29,30], insecticidal [31,32], antidepressant [33,34,35], food preservative [36], analgesic, and anti-inflammatory [37] properties, are recently gaining popularity. Therefore, the latest research paid enormous attention to nanofibers’ application with antimicrobial agents that will be used in tissue engineering, drug delivery, food, cosmetics, and wound healing. Pant et al. prepared salicylic acid/polyurethane nanofibers, showing good mechanical qualities, excellent biocompatibility, and antibacterial action, thus having considerable promise for various future health applications, including tissue engineering, injury treatment, and medicines [38]. Similarly, they also fabricated electrospun carbon nanofibers loaded with titanium dioxide/ zinc oxide nanoparticles (TiO_2_/ZnO NPs), which demonstrated a strong photocatalytic activity for the decomposition of methylene blue under UV irradiation, and showed good antibacterial properties as well [39]. However, this review article will discuss the application of several essential oils, such as lavender, thyme, cinnamon, peppermint, tea tree, etc., encapsulating composite fibers, and mainly electrospun fibers.

**Figure 1:**
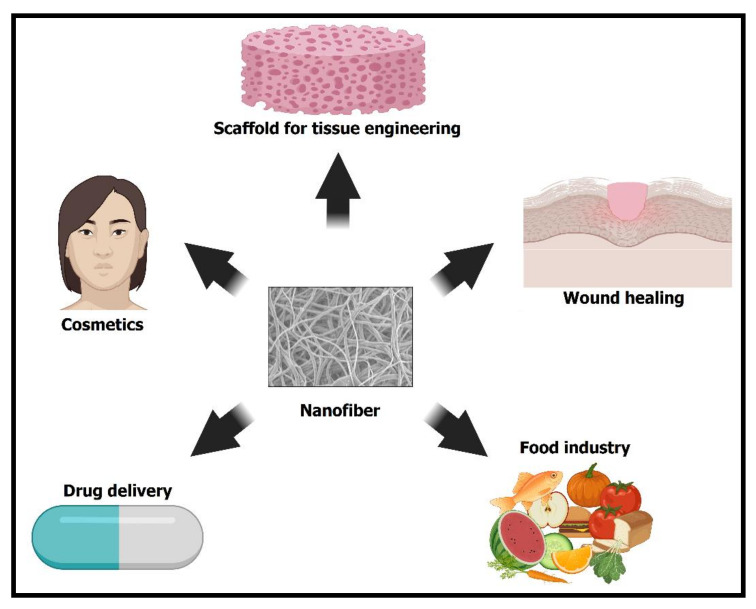
Different applications of fabricated nanofibers in cosmetics, drug delivery, the food industry, wound healing, and hard-tissue engineering.

The application’s focus for the already-fabricated nanofibers will be for wound healing, tissue engineering, cosmetics, drug delivery, and the food industry, as shown in Figure 1. Furthermore, various polymeric nanofibers of sodium alginate, polyurethane, polyacrylonitrile, polyvinyl alcohol, polycaprolactone, polyethylene glycol, silk fibroin, gelatin, chitosan, poly (lactide-co-glycolide), cellulose, etc., which have been used for encapsulation of essential oils, will be discussed in this review article. Ates et al. noticed that nanocomposites made from biorenewable materials, such as cellulose, polylactic acid, silk, gelatin, natural fiber, and oil-based polymer, have been used in a variety of applications such as food, biomedical, wastewater treatment, energy storage, etc. As a result, these nanocomposites can be used in a variety of applications due to their biocompatibility, biodegradability, and thermal and mechanical properties in the near future [40]. In this vein, essential oils’ encapsulation into polymeric fibers has opened new ways for wound dressings, scaffolds for tissue engineering, and active food-packaging structures with progressed antimicrobial activities. Moreover, the nanofibers can supply naturally derived chemical compounds in a controlled manner and prevent their degradation. Likewise, the produced fibers discussed in this review article are supposed to maintain stability and integrity, prolonging a product’s shelf-life while bypassing biofilm creation. These nanofibers also show remarkable antimicrobial and antioxidant activities. Therefore, they have a bright potential for pharmaceutical, cosmetic, and food-packaging operations. This review article summarizes the research done so far on the encapsulation of various therapeutic essential oils in polymeric nanofibers, including lavender, thyme, cinnamon, tea tree, clove, peppermint, rosemary, and others. The ultimate goal is to build practical membranes for biomedical and food-packaging uses (Table 1).

## 2. The Essential Process of Electrospinning to Form Nanofibers

**Figure 2:**
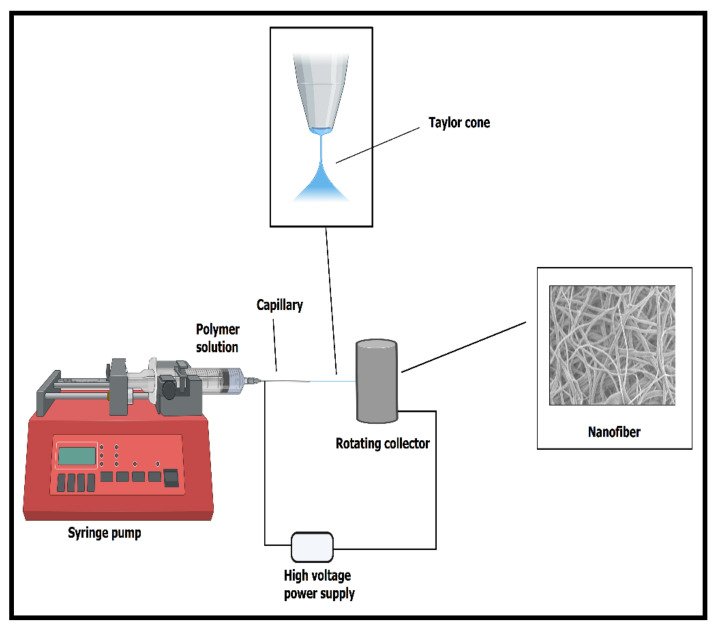
Simple representation of the formation of the Taylor cone and the electrospinning setup to form nanofibers using a high-voltage power supply.

The electrospinning process is a simple and versatile method used to fabricate nanofibers with a diameter in the range of the submicron to the nanometer scale [58]. Nanofibers can be processed from various materials, such as synthetic and natural polymers, ceramics, and/or composites [10]. Compared to ceramics and/or their composites, polymers’ benefit is that they can be formed into nanofibers using various nontoxic solvents, or can be electrospun after blending with other polymers (owing to appropriate viscosity) [59]. The ultrafine fibers fabricated by electrospinning have drawn much attention due to their prominent properties, such as high porosity and large surface-to-volume ratio. This has resulted in superior mechanical properties if we compare the nanofiber mats with polymeric films of the same thickness [58,60]. These nanofibers have enormous applications in tissue engineering, drug delivery, stem-cell differentiation, and wound dressing because of their aforementioned versatile nature and exceptional qualities [61,62,63]. Generally, drug-filled nanofibers using a drug and a polymer solution are considered sufficient; however, the release of the drug from the nanofiber is problematic due to the initial burst release. Therefore, the use of a core-sheath-shaped nanofibers fabricated by coaxial electrospinning offers a promising method of managing the initial burst release [64]. In brief, in the electrospinning process, as illustrated in Figure 2, one end of the electrode is attached to the spinneret’s tip, and the other end is connected to the high-voltage power supply. This power supply generates a positive charge that is attached with the spinneret using an alligator clip (typically more than 5–40 kV). The collector (flat or round exterior) is grounded with a negative charge with high voltage. When the charges generated inside the polymer solution reach a critical point, the solution forms a Taylor cone at the capillary tip and is forced out from its end [65]. The polymeric solution’s jet loop becomes longer and thinner when heading close to the oppositely charged collector. Eventually, it solidifies while depositing on the collector due to solvent evaporation during its travel. The collector onto which fabricated micro- or nanofibers are deposited are placed some distance away (usually 5–20 cm) from the spinneret [66]. The polymers used for electrospinning must be soluble in a desirable solvent. A single-phase homogenous solution is attained, which is very important for forming defect-free fibers. By appropriately controlling the solution parameters, such as conductivity, viscosity, dielectric constant, and surface tension; and the processing parameters, such as flow rate, polymer concentration, the distance between the tip and collector, temperature, and humidity, fibers with desirable morphologies and diameters can be produced [67,68,69]. The solution concentration should be proper (not too high or too low) to form smooth and properly defect-free fibers. A lower flow rate generally results in thin nanofiber preparation because the jet gets sufficient time to elongate and polarize. However, for fiber formation without dripping and beads, i.e., smooth fibers, a maximum distance between a collector and spinneret tip is usually desired [59].

## 3. Essential Oils That Are Used to Modify the Nanofibers for Advanced Delivery

**Figure 3:**
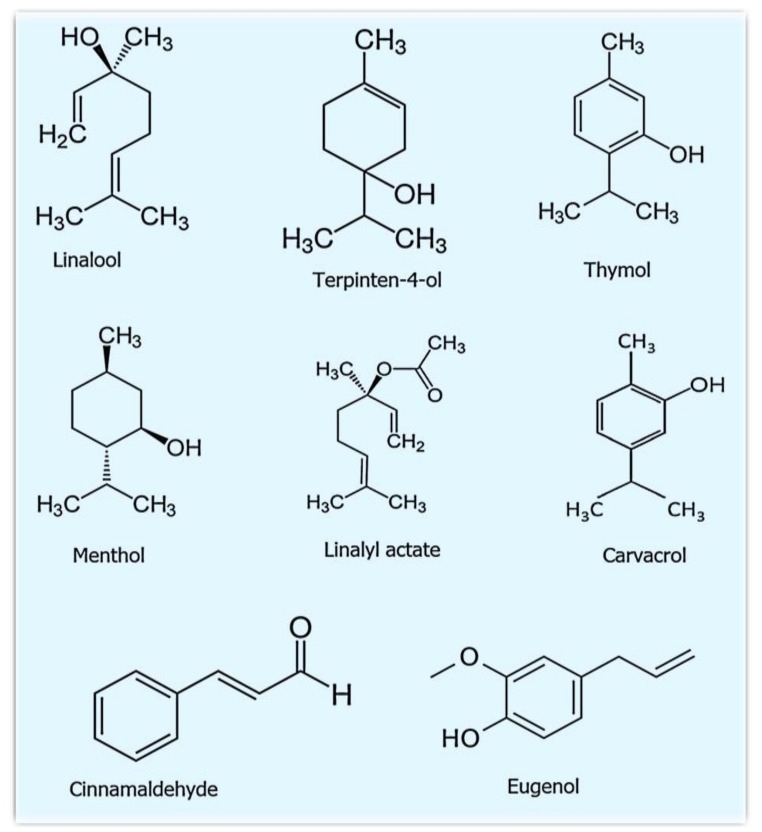
Structures of some of the important constituents of essential oils (reproduced from Wikipedia) via created common licenses.

Depending on the source, essential oils are highly volatile, colored, and/or transparent in nature. They are considered natural products with a strong smell and are generally produced by aromatic plants as secondary metabolites [70]. Although they are the secondary metabolites, their susceptible nature can be degraded quickly under the influence of oxygen, light, and moderate temperature fluctuations. These oils represent a significant part of conventional pharmacopeia (i.e., the official publication of medicinal drugs). Generally, the essential oils are extracted from flowers (rosemary, chamomile, and lavender), fruits/berries (black pepper, juniper berry, and May chang), buds (clove), leaves (thyme, eucalyptus, and rosemary), roots (ginger, angelica), seeds (coriander, cardamom, and fennel) and bark (cassia and cinnamon) of aromatic plants. Furthermore, these essential oils are also deposited in glandular trichomes, cavities, and plant secretory cells [36]. The natural distillate of essential oils is classified as generally recognized as safe (GRAS) by the US Food and Drug Administration (US-FDA); therefore, it received approval for safety and effectiveness [71]. The oils present in these aromatic plants are known to exhibit some excellent properties, such as antioxidant [28], antiparasitic [29,30], insecticidal [31,32], analgesic, and anti-inflammatory [37], and as food preservatives [36]. The researchers observed that the oregano essential oil (OEO), rich in carvacrol and thymol, is an effective antimicrobial agent against Gram-positive and Gram-negative bacteria such as *Staphylococcus aureus, Escherichia coli*, *Pseudomonas aeruginosa,* and *Klebsiella pneumonia,* as well as against other multiresistant Gram-positive and Gram-negative bacteria. This oil also exhibits antitumor activities against a variety of cancers, such as hepatic, cervical, colon, and breast cancers, with IC50 values ranging from approximatively 8 to 300 µg/mL. In addition, the antibacterial, ant-inflammatory, and antioxidative effects of OEO have been shown to aid certain common skin disorders such as acne, aging, and wound healing [72]. Aromatic plants that are primarily grown in temperate and tropical countries produce essential oils as secondary metabolites that can suppress microbial growth [73,74,75]. The essential oils and their constituents mainly target the cytoplasm and plasma membrane and sometimes change the cell morphology, leading to microbe death. It is worth mentioning that more than 60 individual components are present in essential oils [76,77]. The key ingredients (Table 2) can comprise up to 85%, whereas other components are available in trace amounts [77,78]. The phenolic compounds of essential oils are mainly responsible for their antimicrobial activity [79]. The composition of aromatic oils is enormously altered by gene type (species, cultivar, clone, ecotype), environmental factors (geographical origin, climatic condition, soil composition), and technical factors (cultivation, types of collection, storage of crude material, and processing technique). For these reasons, a plant of the same species but from different conditions can reveal contrasting characteristics and chemical compositions [80,81]. Therefore, few methods are mainly used to extract essential oils, including steam distillation and hydrodistillation. However, steam distillation is the most preferred method, as it does not require any solvent and is faster than other methods. Additionally, steam distillation preserves the pristine quality of oils. Structures of some of the main constituents of essential oils are shown in Figure 3.

## 4. The Rationale of Encapsulation of Essential Oils into Polymeric Nanofibers

Due to their nontoxic behavior, natural polymers are preferred over conventional petroleum-based polymers, as their utilization reduces environmental, health, and food problems [82,83,84]. Even though essential oils have a tremendous capability, their utilization has still been restricted because of expensive production, slight vapor pressure, huge volatility, low residual effect, intense aroma, and toxicity to plants [85,86]. Therefore, essential oils can be integrated into electrospun nanofibers to protect them from rapid degradation and evaporation, boost their stability and solubility, and hide their strong aroma [87]. Electrospun nanofibers, because of their size and high surface area, are often used for encapsulation. They tend to show large-scale capacity for operations demanding the sustained release of active ingredients. Simultaneously, the nanofiber system involving oils displays convenient levels of biodegradability and biocompatibility [88]. Moreover, compared to pure essential oils, essential oils incorporated within the polymeric nanofiber are highly effective in reducing pests, thus decreasing pesticide usage, exhibiting low toxicity toward nontarget organisms, and improving the residual effect of the active ingredients [89]. Some of the significant essential oils demonstrating excellent applications after incorporation into nanofibers are discussed in the following subsections.

### 4.1. Encapsulation of Lavender Oil

Lavender oil (LO) derived by steam distillation from the *Lavandula angustifolia* flowers has varying medicinal effects. It is used for mild burns and mosquito bites because of its anti-inflammatory and soothing activities. It also serves as an antioxidant, decreases anxiety, has anticancer and antimutagenic effects, relieves discomfort, and is ideal for the treatment of central nervous system diseases. To exploit these abilities, Hajiali et al. developed a stable nanofiber-based on sodium alginate (SA) after adding polyethylene oxide (PEO) and pluronic F127 to overpower the substandard spinnability of the aqueous alginate solutions [90]. This study demonstrated that SA-PEO and SA-PEO/LO nanofibers had water-contact angles of 21° to 26° (i.e., they were hydrophilic). This change in water-contact angle is critical to promote exudate absorption and water-soluble drug distribution [91,92,93].

Similarly, these nanofibers had high mechanical strength; hence, difficult to deform and sufficiently elastic to adjust dressing on a curved skin wound. These nanofibers also showed antiproliferation activity against *Staphylococcus aureus*, the most common bacteria that quickly multiply on burn wounds after injury [94,95]. These antibacterial properties were due to the major constituents, i.e., linalool and linalyl acetate, the former being more efficacious [96,97,98]. In vitro cytotoxicity test showed that more than 90% of the human foreskin fibroblasts remained viable when incubated with SA-PEO and SA-PEO/LO, indicating that these nanofibrous samples are nonpoisonous. Furthermore, these results were in total agreement with a previous study of Prashar et al. [99]. They observed that electrospun nanofiber possessed anti-inflammatory activity, as it reduced the mRNA levels and the protein release of both proinflammatory cytokines IL-6 and IL-8. The burn marks and erythema induced by ultraviolet B light were entirely faded away from animal skin after being treated with this nanofiber. This was verified by the decreased number of IL-6, IL-1β, and TNF-α cytokines. The in vivo results on cytokine profiles after ultraviolet radiation were in agreement with the in vitro data. This study confirmed that electrospun alginate nanofiber dressings loaded with LO had antibacterial and anti-inflammatory activity, and hence were beneficial for the healing of damaged skin.

In another work, a polyvinyl alcohol-lysine/lavender oil (PVA-Lys/LO) nanofiber membrane was produced by Sequeira et al. through electrospinning [100]. The nanofiber with ibuprofen (IBP) and Lys controlled the inflammatory phase that occurs after skin injury and enhanced cell adhesion and proliferation at the biomaterial surface. PVA is a synthetic polymer because biocompatibility, hydrophilicity, good chemical resistance, and viscoelasticity are utilized in various tissue-engineering treatments. So, the above facts are the basis of why Lys and PVA were blended in a polymeric solution before the electrospinning. The developed membrane had adequate mechanical strength to tolerate all the mechanical stresses that membranes undergo during the tissue-remodeling process. The manufactured membranes also displayed 54–77% porosity levels, which were preferable for an effective healing process. The membrane prepared in this study showed the ability to discard the surplus exudate during incubation, thus maintaining a good atmosphere for the healing applications [101]. For the proper healing process, the ideal environment can be attained when wound dressings are permeable to moisture and gases. It was observed that the rate of water-vapor transmission of PVA-Lys membrane declined after adding IBP and LO, but it still was better than in commercially available wound-dressing materials. The addition of IBP and LO to the PVA-Lys membrane increased its water-contact angle, leading to moderate hydrophilicity, which is considered advisable to stimulate cell attachment and proliferation at the surface of the membrane. By radical scavenging assay, we can observe that LO containing nanofibers shows the highest antioxidant activity; therefore, it is widely used in food processing. It protects food products against oxidative damage [100,102]. The cytotoxic study of membranes evaluated through the MTS assay revealed that all the produced membranes were harmless to normal human dermal fibroblasts. Overall, the obtained data demonstrated the PVA-Lys electrospun-membrane-based drug-delivery system’s excellent biocompatibility, encouraging the application as a wound dressing.

In another study, Balasubramanian et al. fabricated NaCl-assisted polyacrylonitrile (PAN) nanofiber loaded with LO by the electrospinning technique [103]. Different concentrations of an electrolyte solution of NaCl (0%, 0.1%, and 0.3% *w*/*w*) were added to the polymeric solution to enhance the electrospinning capability. It was observed that with the addition of 0.3% electrolyte solution, the bead formation stopped, the degree of polydispersity decreased, and the superior nanofibers were formed with an average diameter of 88.55 nm [104,105]. Thermal gravimetric analysis indicated that as the LO was encapsulated into the PAN nanofiber, the thermal stability increased [103]. An in vitro antibacterial assay observed that PAN/LO (100 μg) showed a 14–15 mm zone of inhibition against both *Staphylococcus aureus* and *Klebsiella pneumonia* for more than 30 days. Maximum loading capacity and encapsulation efficiency were achieved when 0.3 wt % LO was encapsulated into PAN. When the LO increased, the loading capacity and encapsulation efficiency also increased in the matrix [106]. An in vitro cytotoxicity test performed by MTT assay showed a 90–100% viability of NIH/3T3 fibroblasts, but as the concentration of LO increased beyond 200 mg mL^−1^, the cell viability was reduced. The LO/PAN nanofibers avoided cell damage even at a larger amount of 200 mg mL^−1^. Thus, PAN nanofibers can be used as a promising antibacterial material in various fields, such as biomedical, textile, and water-treatment applications.

**Figure 4:**
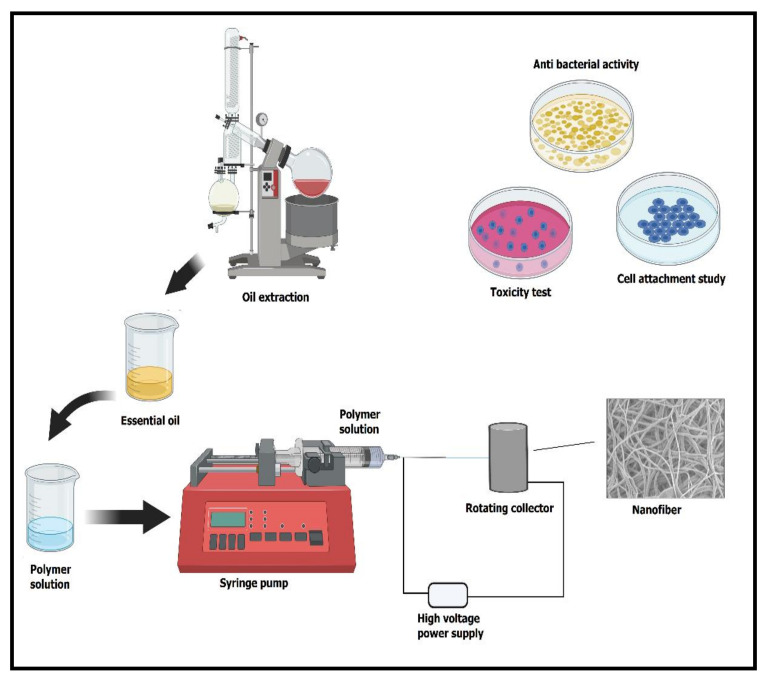
The systematic presentation of the encapsulation of lavender oil in polyurethane nanofibers using the electspinning technique.

**Figure 5:**
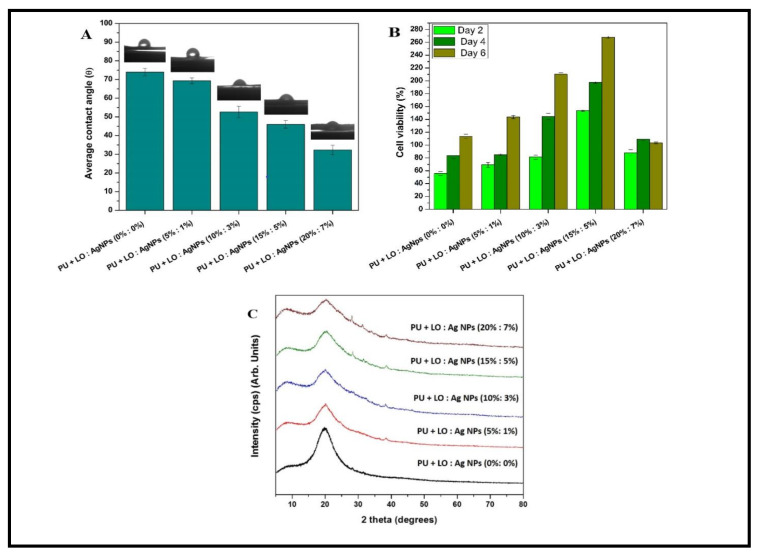
(**A**) The average water-contact angle of pristine nanofibers and composites containing various LO and Ag NPs. (**B**) The cell viability (%) of chicken-embryo fibroblasts cultured on days 2, 4, and 6. (**C**) XRD patterns of nanofibers and composites containing varying amounts of LO and Ag NPs. Reproduced from [107] with copyright permission from Elsevier.

**Figure 6:**
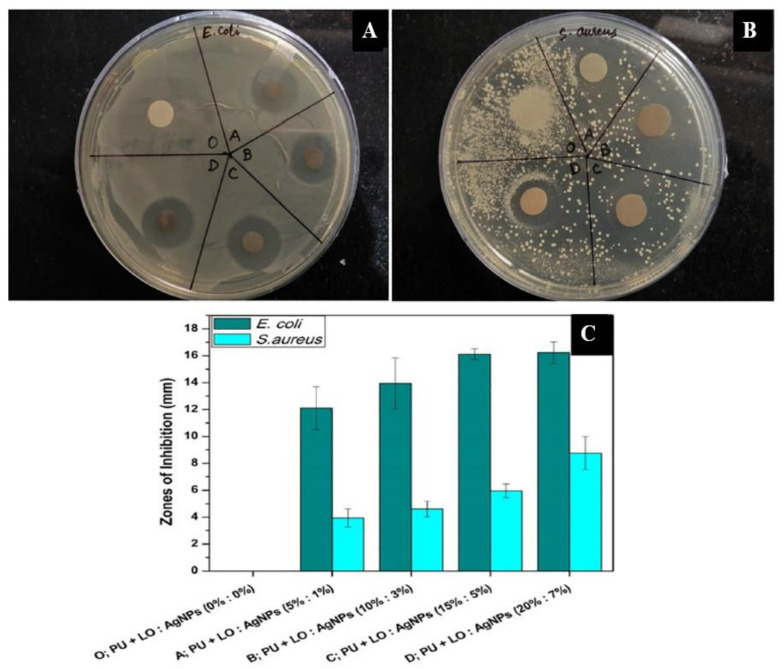
(**A**,**B**) Images of agar plates containing nanofibers with varying concentrations of LO and Ag NPs tested against *Escherichia coli* and *Staphylococcus aureus*. (**C**) Inhibition areas of nanofiber mats and composites were tested by disk-diffusion assay against the above strains, reproduced from [107] with copyright permission from Elsevier

Our group prepared LO and silver nanoparticles (Ag NPs) incorporated with polyurethane (PU) nanofibers for wound-healing applications, as shown in Figure 4 [107]. The literature suggests that PU nanofibers provide several advantages, such as water insolubility, structural support to boost epithelization, noninvasiveness to human cells, and proper pore size for nutrient and gas transfer. However, PU nanofibers’ hydrophobic nature prevents their attachment with the wound, leading to low absorption of exudate and no release of the incorporated antibacterial agents. The addition of LO and Ag NPs (20% and 7%) significantly increased the hydrophilicity of the fiber mats, as illustrated in Figure 5A. At a high concentration of LO and Ag NPs, Ag salt decreases the PU solution’s viscosity and improves the dripping rate [108]. An MTT assay revealed that the viability of a chicken-embryo fibroblast was enhanced significantly with increasing Ag NPs and LO concentrations, up to the limiting concentration of 15% LO/5% Ag NPs, as illustrated in Figure 5B. The X-ray spectra of composites containing different LO and Ag NPs and pristine PU fibers are shown in Figure 5C. The spectrum of the virgin PU fibers was amorphous and did not show any apparent peaks. The diffraction peaks at 2θ of 38.58°, 44.63°, and 64.78°, which correspond to the (111), (200), and (202) crystal planes (hkl values) of Ag, were present in the spectra of the nanofibers that were incorporated with NPs. These results were in total agreement with the results in the literature on PU/Ag NP composites [109,110]. The results of a disk-diffusion assay indicated that nanofibers containing Ag NPs and LO were efficient in inhibiting the growth of *Escherichia coli* and *Staphylococcus aureus,* and increased further as the concentration of LO and Ag NPs increased, as indicated in Figure 6 [111,112,113]. Similarly, Pant et al. successfully established a green strategy to construct an Ag NP-assembled spiderweb-like PU nanofiber mat as a potential antibacterial medium [114]. Pant et al. also fabricated electrospun nylon6/Ag NP composite nanofibers, which showed antibacterial activity against both Gram-negative *Escherichia coli* and Gram-positive *Staphylococcus aureus*; therefore, they can be used in different areas such as wound dressing, water filters, etc. [115]. The outcome obtained from this study suggested that the nanofiber mats containing Ag NPs effectively controlled microbial growth, and could be a promising wound-dressing material.

### 4.2. Encapsulation of Thyme Essential Oil into Nanofiber Scaffolds

Thyme is an aromatic herb from which essential oils are derived. Thyme oil (TEO) is enriched with suitable phenolic active compounds, such as thymol, carvacrol, and eugenol. These are known as natural antimicrobial agents. Nevertheless, these compounds not only provide antimicrobial action against a wide variety of Gram-negative and Gram-positive bacteria, but they also provide thyme’s strong antioxidant ability. Thymol may also be used as an anti-inflammatory agent, which is essential for successful wound healing. Chitosan–gelatin nanofiber with thyme essential oil (TEO) was produced by Vafania et al. via a nozzle less electrospinning technology, an alternative to conventional electrospinning. The aim of this work was to reduce nitrite in sausages [116]. Differential scanning calorimetry analysis confirmed that TEO was successfully incorporated within a nanofiber and was protected against intense heat degradation applied at the time of meat preparation. A 2,2-Diphenyl-1-picrylhydrazyl radical scavenging assay observed that the antioxidant activity of TEO encapsulated inside the nanofiber showed higher antioxidant activity than pure TEO. In this study, undiluted essential oil and essential oil within the chitosan–gelatin nanofiber showed the maximum increase in antibacterial activity against *Clostridium perfringens* due to thymol and carvacrol present in the oil [117,118,119]. *Clostridium perfringens* is an anaerobic, nonmotile, spore-forming, Gram-positive bacterium with rod-shaped morphology that is regarded as a powerful pathogen contaminating meat and meat products. The study showed that the sausages treated with essential oil had a great taste and smell. However, the color and texture did not significantly differ from sausage containing 120 ppm nitrites. Furthermore, the nitrites used as a healing agent to prevent the formation of toxins that destroy nerve tissue can form carcinogenic, teratogenic, and mutagenic N-nitroso compounds. The use of essential oils has enabled us to overcome these drawbacks of toxic nitrites. This research indicates that nanofibers with TEO could be a decent nitrite replacement for meat products. The results obtained from this research revealed that nanofiber-incorporated TEO could be a suitable nitrite substitute for meat products.

Similarly, a great threat to human health and a significant loss in the economy comes from *Campylobacter jejuni,* which also contaminates the surfaces of meat products [120]. Antimicrobial nanofibrous packaging has shown tremendous potential to inhibit the reproduction of *Campylobacter jejuni* on the meat surface. In this regard, Lin et al. prepared antibacterial nanofibers while encapsulating TEO/β-cyclodextrin (β-CD) ε-poly-L-lysine (ε-PLY) (TCPNs) nanoparticles into a gelatin (GEL) polymer using the electrospinning method [119]. The results from this study were that TCPNs containing GEL nanofibers exhibited excellent antibacterial activity against *Campylobacter jejuni* due to the presence of ε-PLY and encapsulation of TEO [121,122]. These disrupt the biological membranes and cause essential protein leakage from the bacteria, the vital substance supporting the viability [123]. The data from this study showed that TCPNs-encapsulated GEL nanofibers have a wide potential in the future for active food packaging. From the sensory analysis, it was seen that there was no significant difference between treatment TCPNs containing GEL nanofibers (TEGNs) and the pure GEL nanofiber. Hence, the TCPNs-loaded GEL nanofibers had a considerable possibility for meat storage without affecting sensory evaluation.

**Figure 7:**
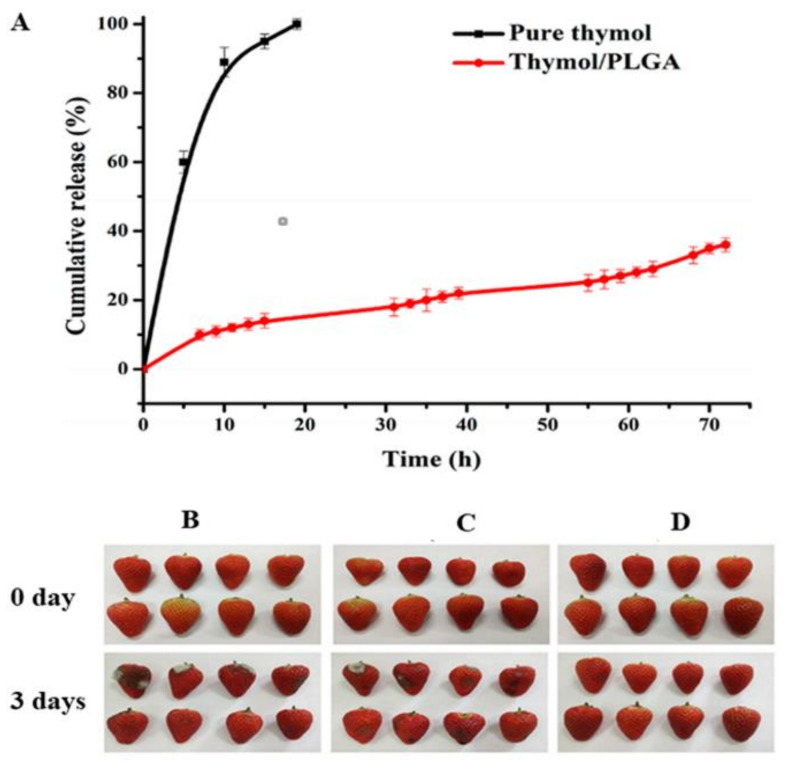
(**A**) Analysis of the release of nanofiber-encapsulated thymol and pure thymol. (**B**–**D**) Pictures of strawberries processed for 0 and 3 days: (**B**) Control, (**C**) strawberries packed with PLGA nanofiber film, and (**D**) strawberries packed with thymol/PLGA nanofiber film. Adapted from [125] with copyrights permission fromfrom the American Chemical Society.

**Figure 8:**
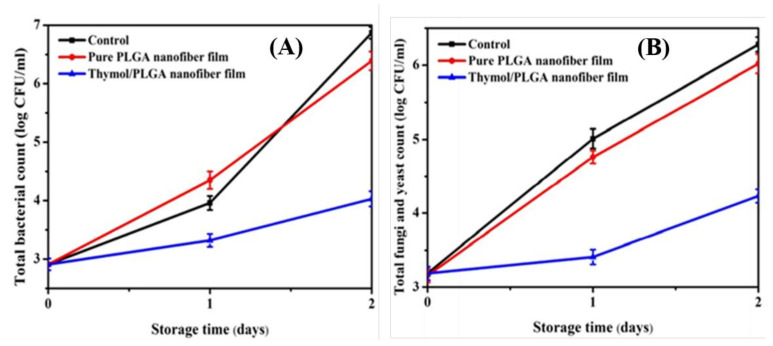
Impact of separate packages on (**A**) the total number of bacteria and (**B**) the total number of fungi and yeast in the strawberries deposited for 3 days at 25 ± 1 °C. Adapted from [125] with copyrights permission from the American Chemical Society.

Thymol is also known to have broad-spectrum antimicrobial activity against an extensive range of microorganisms, such as fungi, yeasts, and bacteria. However, the highly volatile nature of thymol has limited its use for the purpose. The use of nanofibers had enabled us to overcome these drawbacks and attain stability along with the continuous release of essential oils [124]. It can be seen from Figure 7A that unbound thymol evaporated very quickly at room temperature in comparison to thymol incorporated inside the core shell of the nanofiber. In this study, Zhang et al. prepared nanofibers by encapsulating thymol essential oil in a polylactide-co-glycolide (PLGA) polymer via coaxial electrospinning [125]. The coaxial electrospinning method was preferred over single-needle electrospinning. It protected the essential oils inside the core-shell structure formed by this method to play its full role under the protected state [44,126,127]. During the coaxial electrospinning process, the nanofiber film with a core-shell structure was prepared by loading prepared PLGA as a shell solution and thymol as a core solution into plastic syringes. Strawberries covered with the thymol/PLGA nanofiber film showed no evident signs of spoilage, even on the third day. It decreased the bacterial population present on it, as shown in Figure 7B–D. Figure 8A shows the scenario of bacteria, while Figure 8B shows the fungi and yeasts’ position. It was observed that the growth rate of bacteria on strawberries covered with the thymol/PLGA nanofiber film was lesser than on those covered with the PLGA nanofibers and the control group during the storage process. After three days, no visible variation was noticed with regard to the total number of bacterial and fungal growth. This showed that thymol/PLGA nanofibers have excellent potential to extend the storage life and maintain the freshness of the strawberries without disrupting their taste. This new antimicrobial packaging material would have a wide application possibility in food preservation due to its outstanding biocompatibility, environmental friendliness, and better consistent release performance.

In food science, the packaging industry’s motive is to use membranes with low water-vapor permeability, which is a significant step in prolonging food products’ shelf life. This is directly taken care of while using nanofiber membranes as packaging materials. The rationale behind this is the small pore size, which prevents the water permission and allows the sterile gas permission. In this connection, Lin et al. produced thyme essential oil (TEO)/silk fibroin (SF) nanofibers by electrospinning to prevent *Salmonella typhimurium* from infecting fresh poultry meat [128]. Briefly, cold plasma treatment was given to alter TEO/SF nanofibers’ surface properties and increase the release efficiency of TEO from nanofibers [129]. Polyethylene oxide (PEO) was employed to enhance the spinning solution’s viscosity in order to allow the formation of defect-free nanofibers [130]. The results revealed that TEO successfully destroyed *Salmonella typhimurium* within a short period, with minimum inhibitory concentration and minimum bactericidal concentration values of 0.25 mg/mL and 0.5 mg/mL, respectively. The nanofibers obtained displayed good performance by enhancing the mechanical strength. If the concentration of SF was increased to 8:2 (SF: PEO), the water vapor permeability of the membrane decreased remarkably. Thus, cold-plasma-treated nanofibers offer safe and beneficial antibacterial packaging to extend food shelf-life. In brief, the results indicated that plasma-treated TEO/PEO nanofiber-covered chicken and meat samples had great taste and flavor and more attractiveness.

### 4.3. Encapsulation of Cinnamon Oil into Nanofibers

Researchers have used the coprecipitation method for the preparation of cinnamon essential oil (CEO) and β-cyclodextrin (β-CD) inclusion complex into polylactic acid (PLA) polymer to produce antimicrobial PLA/CEO/β-CD nanofilm by electrospinning [131]. The CEO’s thermal stability was enhanced after incorporating it into the β-CD due to the strong interaction between them. PLA/CEO/β-CD nanofilm showed good antimicrobial activity, as it showed the zone of inhibition against *Escherichia coli* and *Staphylococcus aureus* [132,133]. The researchers indicated that pork covered with PLA/CEO/β-CD nanofibers remained preserved for a long time. Further, the PLA/CEO/β-CD nanofilm was harmless and environmentally friendly, which could benefit other meat-based food products when used as active packaging material.

**Figure 9:**
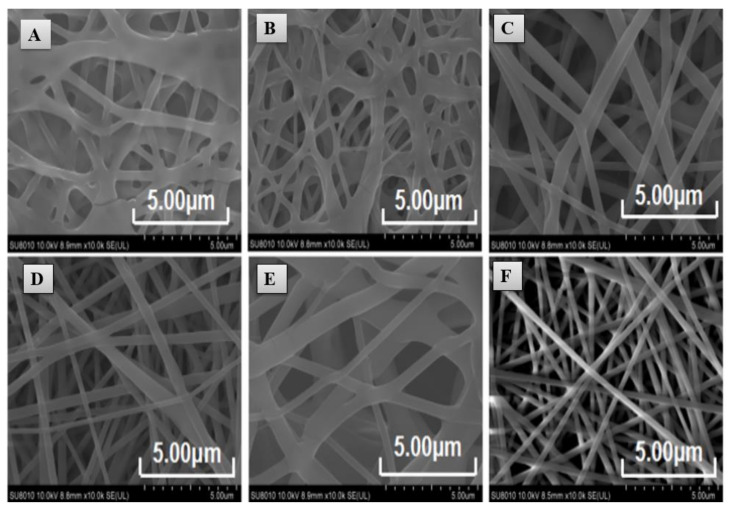
SEM photographs of crosslinking nanofibrous films obtained with the various spinning solutions with and without CEO: (**A**) CPVA, (**B**) CPVA-CEO, (**C**) CPVA-0.5CEO-β-CD, (**D**) CPVA-1.0CEO-β-CD, (**E**) CPVA-1.5CEO-β-CD, and (**F**) PVA. Adapted from [135] with Copyright permission from Elsevier.

**Figure 10:**
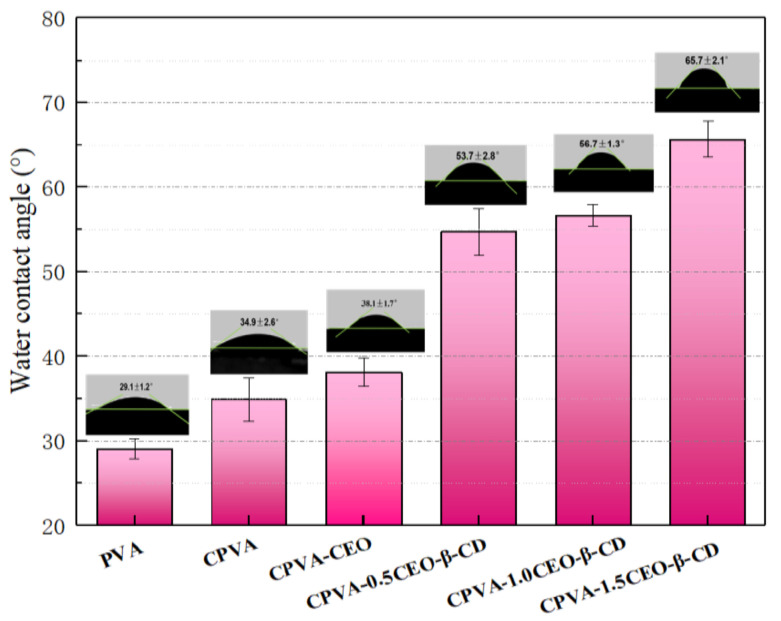
The water-contact angle of various samples. Adapted from [135] with the Copyright permission of Elsevier.

**Figure 11:**
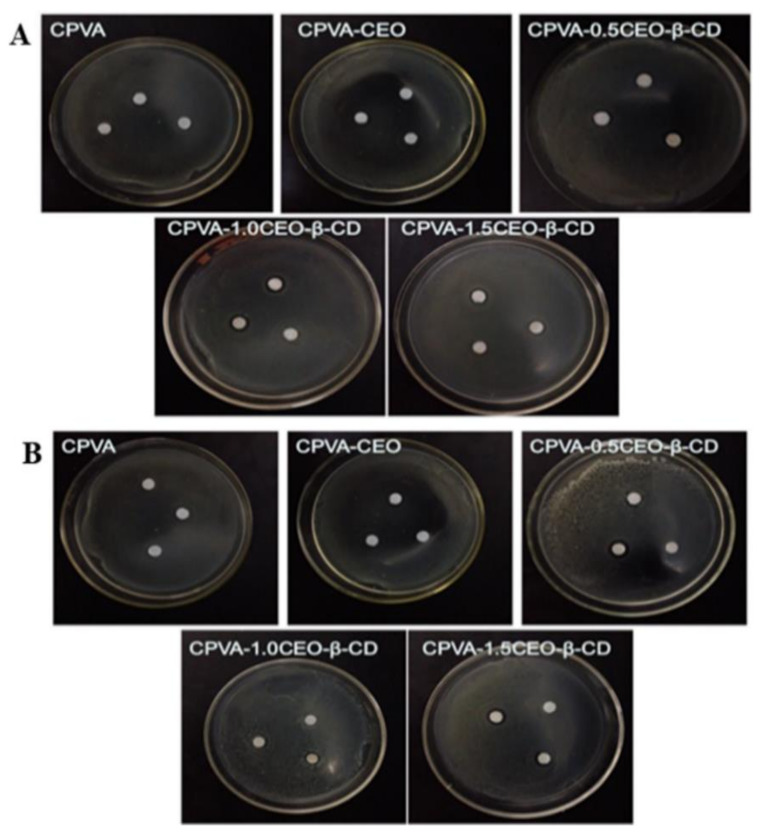
Antibacterial effect against (**A**) *Escherichia coli* and (**B**) *Staphylococcus aureus* by the nanofibrous film. Adapted from [135] with the copyright permission of Elsevier.

Because of brittleness and the absence of defensive tissue, nutritionally rich edible fungi are at greater risk of mechanical damage and microbial spoilage. This leads to losses due to concurrent discoloration, quality deterioration, or even decay of the fruiting body of fungi [134]. In this regard, Pan et al. fabricated an electrospun crosslinked polyvinyl alcohol CPVA/CEO/β-CD nanofibrous film loaded with CEO [135]. Chemical crosslinking and physical welding were achieved by atomizing and fumigating PVA with the aid of glutaraldehyde to reduce its high water solubility [136]. This otherwise limits its application in food preservation despite many advantages such as low price, harmlessness, high chemical stability, excellent film-forming performance, and biological compatibility [137]. CEO was encapsulated into β-CD to reduce its high volatility, water infusibility, and pungent odor [138]. The precise scanning electron microscope images in Figure 9 indicated that the CEO increased the nanofiber diameter when β-CD was available in the structure. The CPVA/CEO (1.5 mL)/β-CD nanofibrous films had a water-contact angle of ≈90°, thus increasing their hydrophilicity, as shown in Figure 10 [139,140,141]. Moreover, Figure 11 shows that when β-CD was added to the PVA/CEO solution, the fibers showed the zone of inhibition against *Escherichia coli* and *Staphylococcus aureus*. This effect was enhanced further as the CEO concentration was increased [142,143]. Thus, CPVA/β-CD/CEO nanofibrous films can slow down mushrooms’ spoilage rate during their storage, demonstrating their potential application in active food packaging.

In another study, Nazaria et al. prepared a unique electrospun crosslinked polyvinyl alcohol-nanophytosome/cinnamon essential oil (CPVA-N/CEO) nanofiber to enhance the bactericidal action and to cut down the toxic effects of essential oils in packing applications [144]. The scanning electron microscope image of CPVA-N/CEO nanofiber displayed homogeneous morphology of the fibers. The CPVA/CEO nanofiber’s water-barrier property was more than that of CPVA-N/CEO due to reducing the overall free amount of nanofiber complex affecting the durability and hydrophobic nature of CEO [145,146]. Another reason for the low water-barrier property of CPVA-N/CEO compared to CPVA/CEO was the amphipathic quality of lecithin in N/CEO and absorption of moisture [147,148]. The addition of N-CEO increased the thermal stability of nanofibers. In addition, the PVA-N/CEO nanofiber was harmless compared to the PVA/CEO nanofiber when evaluated on human colon cancer (HT-29) cells. This may be due to suppressing the CEO’s direct toxicity after encapsulating it into a nontoxic nanophytosome vesicle. The CPVA-N/CEO nanofiber showed higher antibacterial activity against *Escherichia coli* and *Staphylococcus aureus*. CPVA-N/CEO also showed the zone of inhibition against *Pseudomonas aeruginosa*, the leading cause of seafood spoilage at low temperatures. The results showed that with more sustained antimicrobial activity and extending the shelf-life of raw shrimps, CPVA-N/CEO has a high potential for active packaging of shrimps.

Mohammadi et al. investigated the effects of chitosan nanofibers, emulsified cinnamon oil, and cinnamon essential oil-loaded nanolipid carriers on microstructural antibacterial activity and mechanical strength of the resulting bio-nanocomposite films prepared by the casting method [149]. This study introduces a novel eco-friendly bio-nanocomposite in packaging industries for the shelf-life extension of different perishable foods. The nanofibers developed in this study decreased the water solubility and water-vapor permeability of whey protein isolate film by incorporating chitosan nanofibers and cinnamon oil in both forms (emulsified and nanolipid carriers) [150]. Whey protein isolate has high water-vapor permeability, which is due to the presence of hydrophilic groups. This can be diminished by introducing functional groups that decrease the hydrophilic sites or incorporation into chitosan nanofibers, which slows down water molecules’ penetration [151]. It was observed that by incorporating chitosan nanofibers in whey protein isolate films, tensile strength and Young’s modulus increased, but after integrating cinnamon oil in both forms (emulsified and nanolipid carriers), tensile strength and Young’s modulus decreased [152]. The reduced transparency of nanocomposites after incorporating chitosan nanofibers and cinnamon oil resulted in an increase in barrier properties against food spoilers (light, oxygen, heat). The increase in barrier can be favorable for preserving the foods and protecting food against oxidative reactions. The whey protein isolate–chitosan nanofiber composite containing cinnamon oil-loaded nanolipid carriers showed more antibacterial efficiency against *Staphylococcus aureus, Escherichia coli,* and *Pseudomonas aeruginosa* strains than the whey protein isolate–chitosan nanofiber composite containing emulsified cinnamon essential oil.

### 4.4. Encapsulation of Tea Tree Oil

Tea tree essential oil (TTO) is a natural essential oil, a complex blend of different organic compounds derived from *Melaleuca alternifolia*. It exhibits a wide variety of antifungal, antibacterial, antiviral, and anti-inflammatory activity. Its large spectrum of antimicrobial activity is mainly due to its diverse chemical nature, especially its volatile components, e.g., 1,8-cineole, terpinen-4-ol, and terpilenol. *Escherichia coli* O157:H7, one of the leading contagious microbes, boost their numbers immediately on contaminated meat products, such as beef, because of higher moisture and rich protein content [153]. Moreover, *Escherichia coli* O157:H7 can cause serious ailments such as hemorrhagic colitis [154]. So, to control the possible hazard of meat and meat products, Haiying et al. prepared plasma-treated polyethylene oxide (PEO) nanofibers incorporated with tea tree oil (TTO)/β-cyclodextrin inclusion complex (β-CD-IC) by electrospinning [155]. The plasma-treated PEO nanofiber membranes containing TTO/β-CD-IC could extend the shelf-life of beef, thereby suggesting a role as a possible application in active food packaging. The researchers produced nanofibers with a smooth and uniform surface morphology. The results indicated a positive relation between release efficiency and antibacterial activity. After giving plasma treatment to PEO nanofibers, the release efficiency of TTO exceeded, and the antibacterial activity of PEO nanofibers also was enhanced accordingly. For this reason, plasma-treated TTO/β-CD-IC PEO nanofibers showed a higher bactericidal effect than TTO/β-CD-IC PEO against *Escherichia coli* O157:H7 [156,157,158]. Plasma-treated TTO/PEO/β-CD-IC nanofibers at 4 °C, 12 °C, and 25 °C after storing for 7 days showed improvement in color, flavor, appearance, and overall acceptability of beef samples compared with the control and plasma-treated nanofibers at 35 °C [159].

Lee et al. fabricated tea tree essential oil (TTO)-loaded polyurethane (PU) nanofibers by electrospinning for packaging applications [160]. From the mechanical test results, it was observed that as the amount of TTO oil increased, the strength and strain values also improved. PU nanofibers containing 5% TTO showed more significant in vitro antibacterial activity against *Escherichia coli* and *Staphylococcus aureus* than control PU nanofibers [161,162,163]. The cell counting kit-8 assay performed on NIH-3T3 cells revealed that each sample’s cell proliferation with different TTO contents was similar to the control group. Hence, the fabricated nanofibers with TTO were safe for the human body, and could be applied for packaging purposes. To extend the shelf-life of fruits, it is essential to estimate the concentration of CO_2_ inside the packaging, one of the primary components of accepted internal air. It was seen that PU containing 5% of TTO reduced the amount of CO_2_ in fruit packaging, and thus maintained the proper environment for fruits. As the amount of TTO increased in the PU nanofibers, hydrophobicity was slightly lowered, but did not affect the nanofibers’ general hydrophobic nature. Thus, the TTO nanofibers could assist in stopping water from entering inside. The results indicate that tomatoes remained fresh at 30 °C for at least 14 days when they were carefully wrapped with PU–TTO nanofiber.

The antibacterial, anti-inflammatory, antiviral, and antifungal properties of tea tree essential oils can be retained by adding them into biodegradable films [164,165]. In a study by Silveira et al., cassava starch–glycerol reinforced with cellulose nanofibers from *Pinus* sp. nanofiber cellulose and TTO were fabricated by the casting method [166]. The nanofiber cellulose nanocomposites were added to improve these films’ mechanical characteristics [167,168]. Ecologically friendly biopolymers were developed to replace petroleum-derived conventional plastics, which remained in the surroundings for years without being reused or proper disposal [166,169,170]. It was observed that a small concentration of TTO (0.08%) increased the tensile strength due to the crosslinking effect between matrix and oil, while the elongation rate was reduced. However, the value of TTO above 0.08% experienced the opposite situation, except for the low level of nanofiber cellulose concentration when the elongation percentage was even lower. The films containing TTO had a water-contact angle >65°, and therefore were considered as more hydrophobic than control films with nanofiber cellulose and films without the TTO and nanofiber cellulose. The low water-contact angle of control films with nanofiber cellulose and films without the TTO and nanofiber cellulose was due to the cassava starch amylases’ chemical affinity with water [171,172]. Actually, the cellulose fibers were less hygroscopic than starch, which led to the weakening of the amylaceous matrix hydrophilicity, and therefore the film solubility [173]. Despite the oils’ lipidic characteristics, the solubility of films with TTO was somewhat more substantial than the solubility of control films (nanofiber cellulose). This could be related to a decrease in the matrix’s molecular interactions in which the disorder may increase component solubility [174]. Terpinene-4-ol components present in TTO with higher antibacterial activity could be responsible for raising the bacterial cytoplasmic membrane’s permeability and promoting microbial cell damage [175,176,177,178]. The results derived from this research suggest using cassava starch–glycerol with nanofiber cellulose film to cut down the application of synthetic polymers and maintain the active properties of TTO. This film has a bright potential for cosmetic, pharmaceutical, and food-packaging operations.

### 4.5. Encapsulation of Peppermint Oil

Peppermint essential oil (PO) is primarily obtained from the leaves of *Mentha piperita*, a medicinal plant belonging to the Lamiaceae family. It is commonly distributed in the world’s temperate zones, primarily throughout Europe, North America, and North Africa. Menthol, menthone, menthofuran, and menthyl acetate are the main components of this oil. Other bioactive compounds in PO include polyphenols, flavonoids, tocopherols, caffeic acid, choline, carotene, and tannins. It has been used to treat tumors, sore throat, indigestion, fatigue, colds, and cramps, owing to its different bioactive ingredients. It also possesses certain biomedical properties such as antibacterial, antiviral, and antioxidant. Unalan et al. fabricated poly(ε-caprolactone) (PCL) electrospun mats loaded with different concentrations of PO (1.5, 3, and 6% *v/v*) [179]. This study showed that the morphology of the electrospun fiber mats was smooth, uniform, and bead-free following the PO’s integration into the PCL solution. Raman spectroscopy and GC–MS analysis confirmed the presence of 1, 8-cineole, menthol, and menthone, the main components of PO [180,181], after observing the peaks of these different components in the fiber [182,183,184,185]. The PCL electrospun fiber mats’ water-contact angle was 104 ± 8°, although the PO inclusion marginally declined the contact angle of water to 98 ± 5°. This small reduction of the water-contact angle with the addition of PO might be due to the combination of H_2_O molecules with the oxygenated group present in this oil’s chemical structure [186]. The encapsulation efficiency of PO into PCL and its antibacterial activity against *Staphylococcus aureus* and *Escherichia coli* improved as the concentration of this oil increased [11,49,74,177,187]. It was also observed that the PCL nanofiber was harmless to human dermal fibroblasts. However, there was no significant difference between PCL fibers consisting of different PO concentrations. Therefore, PO-incorporated PCL nanofibers prepared by electrospinning are considered promising candidates for wound-healing applications.

Jaganathan et al. developed electrospun polyurethane (PU) nanofibers loaded with PO and copper sulfate (CuSO_4_) for wound dressing [188]. PU was used because of its excellent mechanical and ultraviolet stability, biocompatible and biodegradable behavior, and oxidative and thermal stability [189,190]. CuSO_4_ was incorporated to form an ideal scaffold, as it helps to attain the fiber diameter and wettability beneficial for the growth of fibroblast cells. It also has the antimicrobial activity to suppress the invasion of microbes, which is necessary for wound healing [191]. Atomic force microscopy analysis revealed that the PO/PU/CuSO_4_ nanofibers showed a smaller fiber morphology, resulting in a smooth surface essential for adherence and the growth of fibroblast cells [189,192,193]. In addition, the satisfactory porosity assisted in efficiently delivering nutrients and eliminating waste for superior attachment and growth [188]. The addition of PO improved the water-contact angle of PU, while the addition of CuSO_4_ to the PU/PO reduced the water-contact angle. However, the polymer matrix’s wettability improved, making it suitable for fibroblast adhesion [194]. After adding PEO and CuSO_4_, the tensile strength of the neat PU increased. The presence of PEO and CuSO_4_ in the PU matrix deferred the time of blood clotting, which is advantageous for wound healing, as identified by an activated partial thromboplastin time and prothrombin time assay [195]. An MTS assay found that human dermal fibroblast cells were firmly attached and actively proliferated in all electrospun membranes compared to the control plates. It was also observed that the phenolic components assisted in preserving the fibroblasts against oxidative stress caused by hydrogen peroxide, thereby resulting in cell proliferation and migration [196,197,198]. Accordingly, the prepared wound-dressing composite was based on PU/PO and PU/PO/CuSO_4_ due to their superior physicochemical and biocompatibility qualities, which rendered these fit for wound healing.

### 4.6. Encapsulation of Clove Essential Oil

Clove oil (CLO) is isolated from *Eugenia caryophyllata,* an aromatic flower bud made up of caryophyllene, eugenol, and other compounds such as benzyl alcohol. Owing to its therapeutic properties such as antioxidant, anti-inflammatory, and antimicrobial activities, its key components have been commonly used for decades. Even though it possesses extensive antimicrobial and antioxidant activities, CLO is unable to inhibit the growth of bacteria in the long term due to its volatility and instability [199,200]. CLO’s antibacterial activity is due to its three main compounds: eugenol, eugenyl acetate, and β-caryophyllene. Cui et al. developed CLO-loaded chitosan nanoparticles (CNPs) to enhance their stability and long-term antibacterial activity [201]. The morphology of electrospun CLO@CNP-loaded gelatin (GEL) nanofibers was thin and smooth. These nanofibers were stable and had a uniform diameter [202,203]. Increasing the concentration of CLO@CNPs, GEL, and treatment time improved the in vitro antibiofilm effect of *Escherichia coli* O157:H7 and reduced the population of biofilm formation up to 99.99%. The population of *Escherichia coli* O157:H7 biofilm on cucumber also decreased when tested at 4 °C and 12 °C for four days. However, at 25 °C and 37 °C, the antibiofilm effects of GEL/CLO@CNPs nanofibers were not satisfying because of the faster growth of *Escherichia coli* O157:H7 at a higher temperature. The sensory evaluation results indicated that the GEL/CLO@CNPs nanofiber treatment could maintain the color and flavor of cucumber well for more than four days, compared with the control groups. The overall results indicated that the GEL/CLO@CNPs nanofibers had good stability and antibiofilm activity and could have food-packaging applications.

Another study was conducted by Unalan et al. to design electrospun antibacterial poly(ε-caprolactone)-gelatin/clove essential oil (PCL-GEL/CLO) nanofiber mats for wound healing [204]. The nanofibrous mats could cover the wound area, and could maintain a proper moist environment and antibacterial properties to prevent wound contamination [188,205,206,207,208]. Glacial acetic acid was used as a solvent, as it is nontoxic [209,210]. Smooth, uniform, bead-free, and thin (41 to 300 nm) PCL nanofiber mats encapsulating GEL and CLO were obtained. The inclusion of CLO decreased the water-contact angle, making the PCL-GEL/CLO6% nanofiber mat more hydrophilic, which is desirable for wound-healing applications [211]. In vitro biocompatibility tests showed that CLO-loaded PCL-GEL nanofiber mats were harmless to normal human dermal fibroblasts. On the other hand, after adding CLO to PCL-GEL/fibers, they showed antibacterial activity against *Staphylococcus aureus* and *Escherichia coli* [207,208,209,210,211,212]. Thus, these CLO-loaded PCL-GEL nanofiber mats may have implications in wound-healing operations and can be treated as a promising biomaterial to restrict bacterial infections without using antibiotics.

### 4.7. Oregano Essential Oil

*Oreganum vulgare* L. is known for its essential oil, called oregano essential oil (OEO), a popular compound with proven anticancer, antioxidant and antimicrobial activity. These activities are mainly attributed to two phenols: carvacrol and thymol. Carvacrol, a significant compound, was found to be potentially helpful in tackling free-radical-mediated injuries and saved DNA damage due to its ability to increase the antioxidant level along with the anti-lipid peroxidative activity. Rehman Khan et al. developed OEO-loaded electrospun nanofibers of poly (l-lactic acid-co-e-caprolactone)/silk fibroin (PLCL/SF) to prevent tumor recurrence after surgical removal [213]. The inclusion of OEO increased the mean pore diameter and porosity [214]. This high-porosity nanofiber helps in transporting nutrients and oxygen to the inner surface, absorbing wound exudates from the wound surface and hence lowering the risk of infection [215,216]. The nanofibers, which provide tensile strength and flexibility in all conditions, were an ideal scaffold for wound healing. The developed nanofibers had an excellent tensile strength (1–32 Mpa) and elongation at break (17–207%), appropriate for skin-tissue applications [217]. A DPPH assay revealed that OEO worked through oxidation of reactive oxygen species (ROS) [218]. Cell counting kit-8 and calcein assays on the mouse 4T1 breast tumor cell line revealed potent antiproliferative activity attributed to the carvacrol and thymol present in OEO. The results obtained from this work confirmed that this fiber is beneficial in preventing tumor recurrence after surgical removal.

Certain readily available sustainable biopolymers, e.g., starch, have incorporated antimicrobial agents to provide microbiological stability to foods [219,220]. Still, they have not been widely used in the packaging industry to replace conventional petroleum plastic, mainly because of their low mechanical, barrier, and technological properties [221,222,223]. Therefore, nanocomposites that exhibit better mechanical, thermal, optical, and barrier properties than pure polymers were incorporated into biopolymer films to overcome these limitations [224,225,226]. In a study by Aguilar-Sanchez et al., the antifungal nanocomposites (2% of bentonite or halloysite) of edible starch films containing Mexican OEO were developed by the casting method [227]. As the concentration of essential oil increased, brightness decreased for both bentonite and halloysite, making them cloudy, which resulted in smoother surfaces that influenced light diffraction [228,229]. Starch polymer thickness enlarged with the incorporation of OEO and nanocomposites [230]. The oxygen and carbon dioxide permeability were elevated by bentonite/OEO fibers, and for halloysite films, carbon dioxide permeability declined as the OEO amount increased. Composite starch/bentonite or starch/halloysite fibers present a fibrous like design, reduce pore density, and expand pore size after adding OEO. Generally, Mexican OEO containing edible starch fibers had an excellent fungicidal effect. Mexican OEO was remarkably effective against *Aspergillus niger,* which was inhibited by both types of films at a concentration of 1% but against *Rhizopus* species. Halloysite films were more effective than bentonite at a 1% concentration of OEO [231,232]. On the contrary, the Fusarium species was the most resistant microorganism; films with bentonite and OEO could only inhibit it at 2% [220].

### 4.8. Rosemary Essential Oil

Rosemary (*Rosmarinus officinalis* L.) essential oil is commonly used for food and medicinal purposes due to its anti-inflammatory, anticancer, fungicidal, antioxidant, and antimicrobial activities, mainly owing to its flavonoid and terpene content. Alizadeh-Sani et al. prepared whey protein isolate/cellulose nanofiber/titanium dioxide/rosemary essential oil (WPI/CNFs/TiO_2_/REO) bio-nanocomposite film by the casting/evaporation method [233]. The transparency value observed from 400 to 800 nm revealed that its transparency was reduced after encapsulating the TiO_2_ and REO into WPI. Therefore, the highest clarity was of pure WPI [234,235]. Furthermore, the WPI/CNF film-transparency value was lowered slightly by REO, but it was not notable. The addition of a higher concentration of TiO_2_ and CNFs to pure WPI enhanced WPI films’ thickness. After encapsulating a higher concentration of CNFs, the moisture absorption value increased, but TiO_2_ and REO incorporation induced the opposite outcome [235,236]. To preserve consistency and minimize moisture transfer between the ambient atmosphere and the product, the packaging material’s water-vapor permeability should be minimal. It was observed that the encapsulation of CNFs, REO, and TiO_2_ decreased the water content, water-vapor permeability, and water solubility of WPI films [237,238]. WPI/CNF 7.5% films blended with TiO_2_ nanoparticles and REO presented more considerable mechanical strength and antioxidant activity. The WPI/CNF 7.5%/TiO_2_ 1% REO2% composite film showed the larger inhibition zone against *Listeria monocytogenes, Staphylococcus aureus, Escherichia coli* O157:H7, *Pseudomonas fluorescens*, and *Salmonella enteritidis* [239]. Because of the excellent distribution of the TiO_2_ and REO into the WPI/CNFs matrix, the films prepared in this study showed better morphological features and good antioxidant and antibacterial activity. They enhanced physicomechanical properties, which are the significant properties needed for packaging applications.

In another study, Amjadi et al. produced electrospun zein/k-carrageenan (KC) nanofibers after incorporating 0.5% and 50% *w*/*w* of zinc oxide nanoparticles (ZnO) and rosemary essential oil (REO) [240]. The SEM image showed that after encapsulating ZnO nanoparticles in zein90/KC10/REO nanofibers, a bead-free homogeneous morphology was observed, indicating no adverse consequence of REO on the nanofiber morphology, which was improved by the ZnO nanoparticle encapsulation. The zein polymer tensile strength, Young’s modulus, elongation at break, water-contact angle, and thermal stability (revealed by DSC) were enhanced by incorporating the KC, REO, and ZnO nanoparticles. An increment in the mechanical properties, thermal stability, and hydrophobic nature of zein after ZnO nanoparticles and REO fillers was due to the hydrogen bonds between zein, KC, and ZnO nanoparticles, which enhanced the polymer chains compactness and crystallinity [241,242,243,244]. The addition of KC and REO increased zein nanofibers’ ability to eradicate free radicals easily, resulting in enhancement of antioxidant activity [245,246,247]. Therefore, the zein90/KC10/REO and zein90/KC10/ZnO nanoparticle nanofibers had the highest DPPH scavenging activity. A biocompatibility assay revealed that in the presence of all nanofibers (zein, zein90/KC10, zein90/KC/ZnO, zein90/KC/REO, and zein90/KC/ZnO/REO), human umbilical vein endothelial cells showed more than 97% of viability, indicated that the nanofibers exhibited no toxicity toward these cells after 24 h. It was found that after increasing the incubation time from 48 h to 72 h, the cell viability decreased. However, it was higher than 80%, which proved that the nanofiber samples had excellent biocompatibility and the capability to support cell proliferation. Furthermore, it was found that the zein-based nanofibers enhanced the viability of human umbilical vein endothelial cells after incorporating the KC into them [248]. Zein90/KC10/ZnO nanofiber displayed limited cell viability after 48 and 72 h since ZnO released Zn^2+^ ions, which induced a homeostasis imbalance, resulting in unsatisfactory cellular responses such as cytotoxicity, damage to DNA, and oxidative stress [244]. However, the cell-viability values were almost similar between zein90/KC10/ZnO/RE and the control samples. This phenomenon could be related to the compensation of the ZnO nanoparticles’ detrimental effect on cell viability by conjunction with REO. Zein90/KC10/ZnO and zein90/KC10/REO nanofibers showed a more significant inhibition zone against *Escherichia coli* and *Staphylococcus aureus* as compared to pure zein (control) and zein90/KC10 nanofibers, which did not show antibacterial activity against these strains [241,242,243,249] Therefore, the zein90/KC10/ZnO/REO nanofibers exhibited the highest antibacterial activity against *Escherichia coli* and *Staphylococcus aureus*. The ZnO produced reactive oxygen species and released Zn^2+^, which increased cell-membrane permeability and interrupted protein synthesis and DNA replication, resulting in the killing of bacteria [244,250]. Due to the presence of antibacterial components in REO, such as carnosic acid, rosmarinic acid, carnosol, and isopropanol, and rosmanol, REO-containing nanofibers showed antibacterial activity [242]. Therefore, the prepared electrospun nanofiber zein/KC/ZnO/REO is considered an essential candidate for packaging purposes to enhance shelf-life and quality of food products.

### 4.9. Encapsulation of Ginger Essential Oil

Ginger essential oil (GEO), produced in tropical and subtropical countries such as India and China, is extracted from *Zingiber officinale*. It is one of the most commonly used spices worldwide, especially in Asian countries, because of its potent antifungal, antimicrobial, and antioxidant activities. The flavor of ginger makes it an excellent condiment and appetizer that is popular in food items. Khaledian et al. evaluated the effects of electrospinning of cellulose nanofiber (CNF) integrated with ginger essential oil (GEO) and citric acid (CA) on the chemical, microbial, and sensory characteristics of barbecued chicken meat [251]. Microbial analysis observed that CNF-GEO-CA showed the highest activity against the main microbes involved in chicken-meat spoilage, such as *Enterobacteriaceae* species, *Pseudomonas* species, total aerobic psychotropic bacteria, lactic acid bacteria, and fungi (yeasts and molds) compared to control and CNF treatments [239,252,253,254]. The microbiological test carried out in this study revealed that the GEO could be considered an effective antimicrobial agent. It enhanced chicken meat’s shelf life by lowering the number of microorganisms present on it [255,256,257]. The GEO antimicrobial efficacy improved when checked on the ready-to-cook chicken after introducing CA and GEO into CNF. This was attributable to CA’s uninterrupted entrance into the bacterial cell, which acidified the cell membrane, denatured the cell’s protein, inhibited metabolic reactions, and eventually caused bacteria to die [258,259]. Furthermore, these substances remained high over time because of the continuous release of these antimicrobials by the polymer matrix into the food surface to suppress microbial growth [260]. The sensory analysis evaluated that color, taste, odor, and overall acceptability of chicken meat were enhanced by wrapping it with CNF containing GEO and CA during the storage.

Silva et al. fabricated ultrafine fibers by incorporating ginger essential oil (GEO) into zein (Z); polyethylene oxide (PEO), and soy protein isolate (SPI) polymers via electrospinning to restrain *Listeria monocytogenes* growth in fresh Mina’s cheese [251]. The GEO showed antibacterial activity against *Escherichia coli* 0157:H7, *Listeria monocytogenes*, *Salmonella typhimurium*, *Staphylococcus aureus,* and *Pseudomonas aeruginosa.* However, in comparison with other strains, *Listeria monocytogenes* were too delicate to the action of GEO and exhibited the largest area of inhibition [261]. For *Escherichia* coli 0157:H7 and *Listeria monocytogenes*, GEO had a minimum inhibitory concentration and minimum bactericidal concentration at 2.3 μL.mL^−1^ and 4.7 μL.mL^−1^. Despite GEO’s content, the ultrafine fibers prepared from the SPI-PEO-Z blend displayed cylindrical shapes with homogeneous morphology without any bead formation after the electrospinning. The average diameter of fibers increased as GEO concentration (3%, 6%, 9%, and 12%) increased. After penetration into polymers, the GEO’s thermal stability improved. The number of *Listeria monocytogenes* on the fresh cheese decreased when the cheese was wrapped with fibers consisting of GEO as compared to cheese covered with fiberless packaging and fibers without GEO. The in situ analysis of the 12% GEO nanofiber revealed that active fibers could be an important and natural alternative for managing *Listeria monocytogenes* in Mina’s fresh cheese during storage. In this report, it was noted that the oil incorporated in the fibers could extend its antimicrobial activity. Besides, the release of the essential oil compounds into the food through volatilization did not involve physical touch, which can reduce the unsatisfactory sensory characteristics that can occur in foods. Therefore, active fibers containing 12% of GEO provided a strong probability of being used to eliminate microbial contamination in food packaging.

### 4.10. Other Less-Common Essential Oils That Are Delivered While Encapsulating Them in Nanofibers

**Figure 12:**
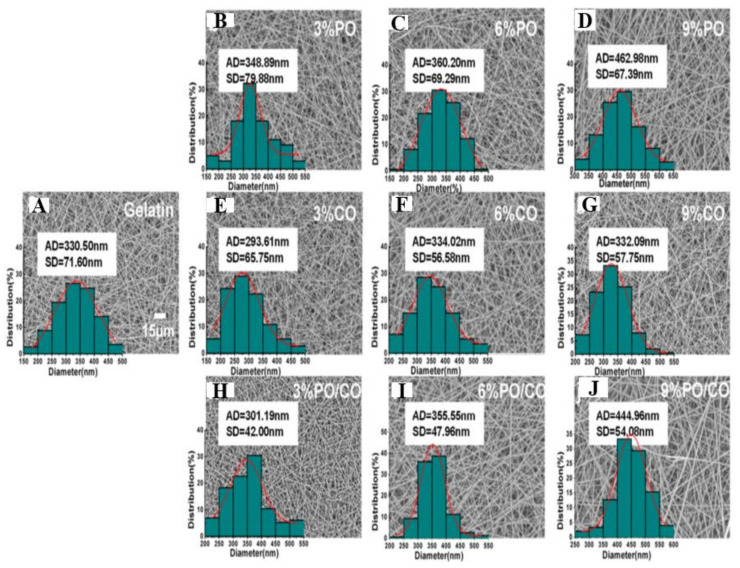
GEL/essential oil nanofiber diameter distribution and scanning electron microscope photos. (**A**) GEL nanofibers; (**B**–**D**) GEL/PO nanofibers with PO ratios of 3%, 6%, and 9%; (**E**–**G**) GEL/CO nanofibers with CO ratios of 3%, 6%, and 9%; (**H**–**J**) GEL/PO/CO nanofibers with PO/CO ratios of 3%, 6%, and 9% (*v/v*), respectively. Standard deviation and average diameter are referred to as SD and AD, respectively. Reproduced from [262] with the copyright permission from American Chemical Society.

**Figure 13:**
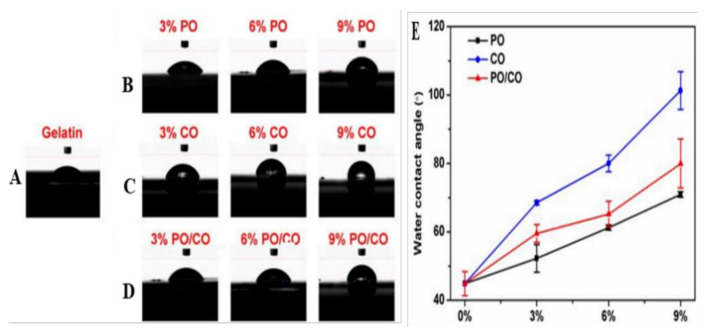
Water droplet test photos on (**A**) GEL nanofibers, (**B**) GEL/PO nanofibers, (**C**) GEL/CO nanofibers, and (**D**) GEL/PO/CO nanofibers. (**E**) Nanofibers loaded with different essential oils’ (blue, CO; black, PO; and red, PO/CO) water-contact angles. Reproduced from [262] with copyright permission from the American Chemical Society.

**Figure 14:**
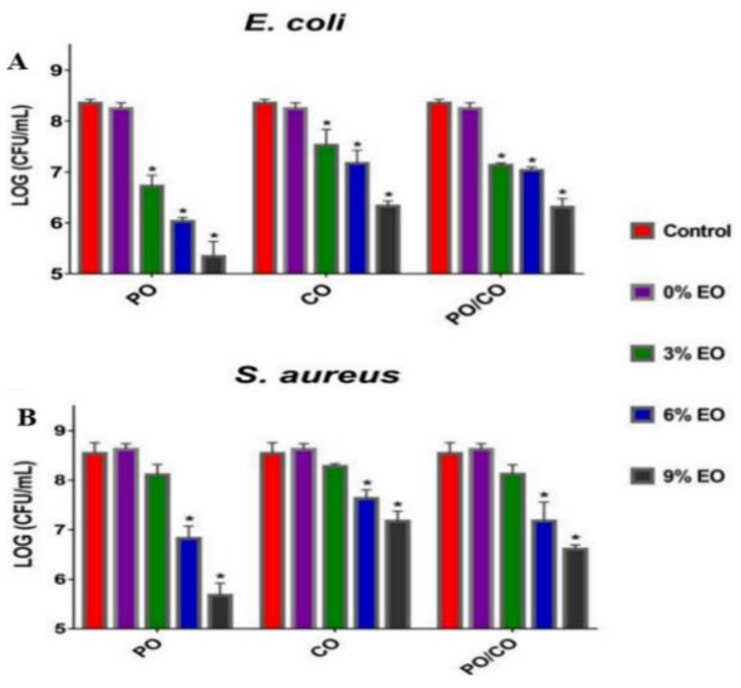
Bactericidal efficiency of Gel/essential oil nanofibers against (**A**) *Escherichia coli* and (**B**) *Staphylococcus aureus*. * *p* < 0.05 against the control group. Results are the mean ± SD (*n* = 3). Reproduced from [262] with copyright permission from the American Chemical Society.

**Figure 15:**
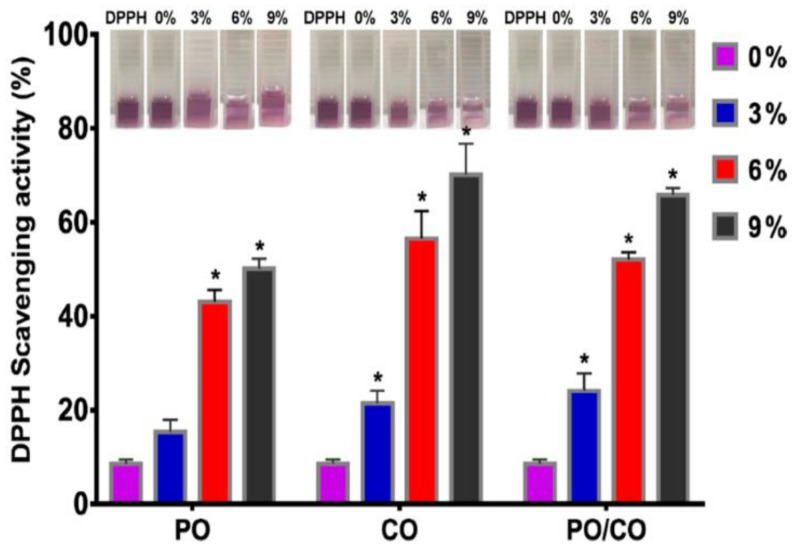
Different concentrations (0%, 3%, 6%, and 9%) of essential oil (PO, CO, and PO/CO)-containing nanofibers’ DPPH radical scavenging activity and the corresponding images of DPPH solution accommodating different nanofibers. * *p* < 0.05 versus the control group. Results are the mean ± SD (*n* = 3). Reproduced from [262] with copyright permission from the American Chemical Society.

Tang et al. prepared two essential oils, chamomile oil (CO) and peppermint oil (PO) loaded gelatin (GEL) nanofibers via electrospinning for implementation in edible-packaging materials [262]. The photographs of the different nanofibers’ diameter distribution and scanning electron microscope images are presented in Figure 12. All groups of GEL/essential oil nanofibers had a smooth and bead-free structure. No apparent deviation was observed from pure GEL nanofibers, indicating the homogeneous distribution and incorporation of the essential oils in GEL nanofibers. With the rise in the concentration of essential oils, the diameter of GEL/essential oil nanofibers was vastly widened, which may be attributed to the increased viscosity of the spinning solution resulting from the inclusion of essential oils [43,263,264]. Incorporating essential oils turn the hydrophilic GEL polymer into a hydrophobic polymer to avoid moisture movement between food and the environment to prolong the food shelf-life [265,266]. Contrastingly, GEL nanofibers with CO were more hydrophobic than those with PO at the same quantity, as indicated in Figure 13 [267,268]. Figure 14 shows that the GEL nanofiber incorporated with essential oils inhibited *Escherichia coli* and *Staphylococcus aureus* growth; this inhibition was seen more against the former strain [269,270,271]. The antibacterial activity also was enhanced as the concentration of essential oils was increased, and in comparison to CO, PO-containing GEL nanofibers exhibited more considerable antibacterial activity against both microorganisms. DPPH radical scavenging activity of the nanofiber loaded with different concentrations of PO, CO, and PO/CO essential oils (0%, 3%, 6%, and 9%) indicated that with the increase of the essential oil amount, the antioxidant activity increased, as illustrated in Figure 15 [272]. The CO-containing nanofibers displayed more excellent antioxidant activity than the PO groups, whereas the GEL/CO/PO nanofibers were mainly intermediate. The MTS assay results demonstrated that nanofibers of GEL/essential oils were harmless to NIH-3T3 cells, increasing their cell viability, which, as shown, made them eligible for edible packaging use [51]. The role of evolved GEL/PO/CO as a promising candidate for edible packaging is reflected in these findings.

The essential oil of *Angelica sinensis* (Oliv.) herb, known in Chinese as Dong-gui, has been shown to have excellent antibacterial and antioxidant activities that could be useful as a possible bioactive ingredient in food packaging. Zhou et al. fabricated gelatin (GEL) nanofibers loaded with different concentrations (0%, 3%, 6%, and 9%) of angelica essential oil (AEO) through electrospinning [273]. In general, as the viscosity increased, the nanofiber diameter also increased due to less stretching of fiber. Moreover, with the addition of electrical conductivity, the diameter was reduced because of more elongation of the fiber. In this analysis, the rise in the AEO concentration also increased the viscosity and the electric conductivity, which led to the final increase in the nanofiber diameter [264]. The best food-packaging material has a high water-contact angle (hydrophobic), antioxidant activity, and antibacterial activity. The GEL nanofiber’s water-contact angle improved as the concentration of AEO increased, which could be related to the hydrophobic nature of AEO [274,275]. Likewise, the increased amount of AEO enhanced the antioxidant activity of GEL/AEO nanofibers, and hence protected the food from oxidative damage. Due to the presence of an E-3 butylidenephthalide component, AEO had outstanding antioxidant properties [276]. The GEL nanofibers with 9% AEO concentration showed the highest bactericidal effect against *Escherichia coli* and *Staphylococcus aureus* as revealed by the shake-flask method [277]. The results demonstrated that the GEL/AEO nanofiber was essential in extending the shelf life of the products.

Chrysanthemum oil (CHEO) is an aromatic oily liquid extracted from chrysanthemum. A perennial herb, chrysanthemum belongs to the family Asteraceae, and may help avoid different ailments, such as coronary artery disease, hypertension, and arteriosclerosis. This oil has immunomodulatory, antibacterial, anti-inflammatory, and antifungal activities, according to previous research. Few studies have referred to CHEO’s antibacterial function and antibacterial process. Lin et al. prepared chitosan nanofiber (CSNF) with CHEO by electrospinning to eradicate *Listeria monocytogenes*, which causes severe deterioration in meat products [278]. This oil destroyed the *Listeria monocytogenes* cell membrane, creating the leakage of cellular substances, such as DNA, proteins, and ATP molecules. It also repressed the respiratory metabolism by suppressing the Embden–Meyerhof–Parnas pathway [279], and harmed the activity of hexokinase, phosphofructokinase, and pyruvate kinase in *Listeria monocytogenes*. The scanning electron microscope images showed the chitosan and its blends with CHEO nanofibers were continuous, smooth, and well-distributed [280]. The inclusion of CHEO into nanofibers enhanced the thickness and moisture content, but decreased the water solubility because of CHEO’s hydrophobic nature. GC–MS analysis showed the release rate of CHEO from the nanofibers, and it was observed that CHEO was released slowly during 15 days of storage at all temperatures (4 °C, 12 °C, 25 °C). Subsequently, as the weather improved, the release rate also rose. The main reason behind the worsening quality of meat during storage was fat oxidation, resulting in a severe loss of flavor and nutritional value. Thiobarbituric acid-reactive substances are commonly used to measure lipid peroxidation products in cells, tissues, and body fluids. It was observed that thiobarbituric acid-reactive substance values of the treated beef samples were lower than untreated after 12 days at 4 °C [281,282]. The results indicated that CHEO/CSNF could minimize the rate of lipid oxidation and preserve freshness, thus prolonging the shelf-life of beef.

## 5. Conclusions

This review article discussed the encapsulation of various therapeutic essential oils such as lavender, thyme, cinnamon, tea tree, clove, peppermint, rosemary, etc., in polymeric nanofibers. The overall aim was to develop functional membranes for biomedical and food-packaging applications. The studies discussed in this review article have shown that the bioactivity of essential oils is preserved by combining them with polymeric solutions and transforming them into nanofibers, particularly by electrospinning. These nanofibers exhibit a fibrous morphology with a large surface-to-volume ratio, high porosity, and appropriate fiber diameter in the nanometer to submicron range. These attributes are desirable properties for the sustained release of active ingredients from the packaging membrane to the food surface. These nanofibers also regulate the quantity and release profile of essential oils present in them. They thus reduce the cytotoxic effects on human cells that some components of essential oils have.

## Figures and Tables

**Table 1:** Nanofiber-incorporated essential oils for diverse applications.

Essential Oil	Polymer	Utilization	Ref
Lavender (LO)	Polyurethane (PU)	After the encapsulation of LO and cobalt nitrate, the mechanical properties of electrospun PU scaffolds improved, and therefore were beneficial for bone-tissue engineering. It was seen that nanofibers were harmless to human dermal fibroblasts analyzed through MTS (5-(3-carboxymethoxyphenyl)-2-(4,5-dimethyl-thiazoly)-3-(4-sulfophenyl) tetrazolium assay.	[41]
Lavender (LO)	Polycaprolactone (PCL)/Polyethylene glycol (PEG)	This LO encapsulating PCL/PEG nanofibers generated through electrospinning showed a zone of inhibition against *Staphylococcus aureus* and *Escherichia coli.*	[42]
Thyme (TEO)	Silk fibroin (SF)/Gelatin (GEL)	SF/GEL nanofibers loaded with TEO and doxycycline monohydrate fabricated by electrospinning showed a larger prevention zone against *Staphylococcus aureus* and *Klebsiella pneumoniae*. In addition, the MTT (3-(4,5-dimethylthiazol-2-yl)-2–5-diphenyltetrazolium bromide) assay revealed that this nanofiber was not harmful to mouse fibroblast L929 cells.	[43]
Thyme (TEO)	Polycaprolactone (PCL)/Polyvinyl alcohol (PVA)	Coaxial electrospun core-shell nanofibers incorporated with TEO showed the highest antibacterial activity against *Staphylococcus aureus* and *Escherichia coli.* Thyme-extract encapsulated nanofibers could be used as potential wound-healing material and are promising materials to treat surfaces that contain pathogenic microorganisms.	[44]
Thyme (TEO)	Potato starch	Thermal stability of TEO encapsulating potato-starch nanofibers fabricated via electrospinning has been upgraded and can be applied in food products or packaging that requires high temperatures during their production. It can also be used as natural antioxidants in food products because 50% of this oil’s total phenolic compounds are retained after applying a thermal treatment of 100 °C. The antioxidant activity of the oil mentioned above is related to its significant content of phenolic compounds.	[45]
Cinnamon (CEO)	Polyurethane (PU)	Electrospinning-fabricated PU nanofiber loaded with CEO and activated carbon inhibited *Escherichia coli* and *Staphylococcus aureus* growth. Thus, the nanofiber air filter was more efficient. This nanofibrous air-filter media can be applied in various areas such as antibacterial fibers, personal masks, and air purifiers.	[46]
Cinnamon (CEO)	Polyvinyl alcohol (PVA)	Fumigant bioassays revealed that CEO inside nanofibers generated using electrospinning was more toxic than free CEO against all stages (e.g., male and female adults) of *Phthorimaea operculella*, one of the most common insect pests of cultivated potato. Accordingly, this nanofiber was effective in protecting horticulture extracts from pests during storage.	[47]
Cinnamon (CEO)	Sodium caseinate (SC)/Cellulose	The SC nanocomposite containing CEO and cellulose nanofiber prepared by the solvent-casting method was found to be successful in prolonging the shelf life and maintaining the quality of dry and oxidation sensitive foods such as nuts, spices, and bread and cereal products.	[48]
Cinnamon (CEO)	Polyvinyl alcohol (PVA)	Biodegradable electrospun PVA/CEO/β-cyclodextrin nanofibrous film showed excellent antimicrobial activity against *Escherichia coli* and *Staphylococcus aureus*. It could effectively prolong the shelf-life of strawberries, thus being applicable in active food packaging.	[49]
Cinnamon (CEO)	Polyurethane (PU)	Nanofibers fabricated by electrospinning were nontoxic, as they enhanced the growth of NIH 3T3 fibroblasts. They inhibited the growth of *Staphylococcus aureus* and *Escherichia coli*. They also reduced the chances of fruit decay.	[50]
Cinnamal- dehyde	Chitosan/ Polyethylene oxide (PEO)	Chitosan/citric acid/PEO nanofiber mats manufactured by electrospinning could act as delivery vehicles for this oil, potentially eliminating *pseudomonas* infections.	[51]
Cinnamon (CEO)	Polyvinyl pyrroli-done (PVP)	This nanofiber prepared by emulsion electrospinning showed good antibacterial effects against *Staphylococcus aureus*, *Escherichia coli,* and *Candida albicans* with 2, 3, and 4 wt % CEO.	[52]
Tea tree oil (TTO)	Chitosan	Electrospinning-prepared nanofiber membrane added with liposome-encapsulated TTO showed the maximum inhibition zone against *Salmonella enteritidis* and *Salmonella typhimurium.* It did not corrupt the sensory properties of chicken meat. Therefore, this nanofiber was conducive to extending the shelf life of chicken meat.	[53]
Peppermint (PO)	Polyethylene oxide (PEO)/Graphene oxide	Electrospinning-prepared nanofibrous mat having cerium oxide and PO exhibited prolonged antibacterial activity against *Staphylococcus aureus* and *Escherichia coli* due to the surface charge of cerium oxide and antibacterial properties of PO. Moreover, the in vitro MTT assay revealed that the nanofibrous mat exhibited low cytotoxicity toward L929 fibroblasts. The histological evaluations demonstrated that this nanofibrous mat accelerated re-epithelialization and collagen deposition, making it a potential candidate to be applied as a wound dressing to prevent skin infections. It has been shown that graphene oxide-filled nanofibrous scaffolds possess a porous structure and can maintain a moist environment around the wound, thereby facilitating the wound-healing process.	[54]
Red thyme/Clove oil (CLV)	Polycaprolactone (PCL)	PCL nanofibers added with CLV and red thyme essential oils prepared by electrospinning could be used as biofilm inhibitive agents on surfaces of biomaterials that are frequently contaminated by *Candida tropicalis*.	[55]
Rosemary (REO)/ Oregano (OEO)	Cellulose acetate (CA)	Electrospun CA nanofiber loaded with REO and OEO had good antimicrobial properties against three common microbial species: the bacteria *Staphylococcus aureus* and *Escherichia coli,* and the yeast *Candida albicans.* This work suggested that OEO was more potent than REO against the three studied microbes, possibly due to the immense microbial role of OEO molecules, such as carvacrol and thymol.	[56]
Ginger (GEO)	Chitosan	Chitosan bio-nanocomposite incorporated with sodium montmorillonite and GEO reduced the contamination and therefore improved the shelf life of poultry meat. This fiber can fulfill consumers’ demand for healthier and less chemically modified food products. It maintained the color, flavor, and PH value, and lessened the microbial contamination of meat products wrapped with this fiber.	[57]

**Table 2:** Chemical composition of essential oils that have been impregnated with polymeric nanofibers.

Essential Oil	Main Components
Lavender	Linalool, Linalyl acetate, Camphor, and Eucalyptol
Thyme	Thymol, Gamma-terpinene, Para-cymene, and Carvacrol
Cinnamon	Cinnamaldehyde, Trans-cinnamyl acetate, Eugenol, and Camphor
Tea tree	Terpinen-4-ol, Gamma terpinene, Alpha-terpinene, and 1,8-Cineole
Peppermint	Menthol, Menthofuran, Menthyl acetate, and Menthone
Clove	Eugenol, Eugenyl acetate, Benzyl alcohol, and Beta-caryophyllene
Oregano vulgare	Carvacrol, Thymol, Beta-fenchyl alcohol, and Gamma-terpinene
Mexican oregano	Thymol, Carvacrol, Cineole 1–8, and Para-cymene
Rosemary	Cineole, Camphor, Alpha-pinene, and Camphene
Ginger roots	Alpha-zingiberene, Geranial, (Z)-citral, and Beta-cedrene
Fresh ginger rhizomes	Alpha-zingiberene, Beta-sesquiphellandrene, Trans-gamma-cadinene, and Geranial.
Chamomile	Alpha-bisabolol oxide A, Chamazulene, *n*-Octanal, and 1,8-Cineole
Angelica	(Z)-ligustilide, E-3-butylidene phthalide, (Z)- β-ocimene, and Gamma-terpinene
Chrysanthemum	Borneol, Beta-silenene, Camphor, and Guaia-3,9-diene
